# A Systematic Survey on Sensor Failure Detection and Fault-Tolerance in Ambient Assisted Living

**DOI:** 10.3390/s18071991

**Published:** 2018-06-21

**Authors:** Nancy E. ElHady, Julien Provost

**Affiliations:** Professorship of Safe Embedded Systems, Technical University of Munich, Boltzmannstraße 15, 85748 Garching, Germany; nancy.elhady@tum.de

**Keywords:** ambient assisted living, sensor failure, fault detection, fault tolerance, smart home

## Abstract

Ambient Assisted Living (AAL) systems aim to enable the elderly people to stay active and live independently into older age by monitoring their behaviour, provide the needed assistance and detect early signs of health status deterioration. Non-intrusive sensors are preferred by the elderly to be used for the monitoring purposes. However, false positive or negative triggers of those sensors could lead to a misleading interpretation of the status of the elderlies. This paper presents a systematic literature review of the sensor failure detection and fault tolerance in AAL equipped with *non-intrusive*, *event-driven*, *binary* sensors. The existing works are discussed, and the limitations and research gaps are highlighted.

## 1. Introduction

According to the World Health Organization, the world’s population percentage of people aged over 60 is expected to double in the next decades to increase from 12% in 2015 to 22% in 2050. This phenomenon, known as Ageing Population, can be already witnessed in high-income countries. This demographic shift will induce new challenges to the countries, e.g., preparing the health care and social systems to deal with higher capacities [1]. Focusing on healthy ageing is an essential investment for facing that shift. Taking care of the elderlies would decrease the chance of further complications to their health status. This can be achieved by providing care in nursing homes or hospitals. However, it is costly and the costs increase greatly if the person needs specialized care due to immobilization or other health problems. A cost-effective alternative is using technology for independent living of the elderlies [2].

Ambient assisted living (AAL) is defined as “the use of information and communication technologies (ICT) in a person’s daily living and working environment to enable them to stay active longer, remain socially connected and live independently into old age” [3]. AAL technologies can monitor the behavior of elderly people at home and provide support whenever required, and hence, improve the quality of life [4]. This would cast some burden away from the family members of the elderlies, decrease the need for qualified caregivers and have a positive impact on the psychological status of the elderlies, as they would live independently at their homes longer and safer [5].

*Smart homes* and *ambient assisted living* (AAL) terms were found to be interchangeably used in scientific articles, however, AAL is a special form of a smart home. AAL tools range between health and activity monitoring tools, wandering prevention tools, and cognitive orthotics tools [6]. The technology of those tools are based on ambient intelligence, a paradigm that integrates technology in people’s environment to help them in their everyday lives by learning and adaptively responding to their behaviour [7]. Researchers are interested in investigating approaches to track the location and the activities of the residents, prompting the residents, discovering the abnormal behavior, and predicting the future activities [8]. Integrating sensors in an unobtrusive intelligent way in the residents’ homes, allow monitoring their activities of daily living (ADL) to track their health status, and to detect early signs of diseases [9].

The sensors used to monitor and locate the resident can be classified into intrusive sensors (e.g., camera, microphone) and non-intrusive sensors (e.g., motion detectors, pressure sensors). In practice, the sensors installed in the inhabitant’s place of residence may produce wrong output, e.g., false positives or negatives. A failure in one of the sensors of the AAL could lead to misleading result in activity recognition, or in location tracking. This can have dramatic consequences to the health of the inhabitant [10].

This survey paper aims to review the research work done in the sensor failure detection and fault tolerance in the presence of sensor failures in AAL systems equipped with non-intrusive binary sensors. The paper is organized as follows; Section 2 provides an overview of sensor failures, Section 3 presents an overview of the typical publicly available datasets used in the reviewed works, Section 4 outlines the methodology used to conduct the literature survey, Section 5 presents the research work found in the survey, Section 6 discusses the reviewed works and Section 7 discusses the status of research and highlights the gaps.

## 2. Background on Sensors Failures in Smart homes and AAL

A *fault* can be defined as an abnormal event that can cause an element or an item to fail, while a *failure* is the termination of the ability of an element to perform a function as required [11]. A fault may or may not lead to failure.

For sensor networks in general, two perspectives for fault type classification in sensor networks was proposed by [12]:

1. Data-centric viewpoint, which is based on the characteristics of sensor readings, e.g., stuck-at and spike.
2. System-centric viewpoint, which describes faults causing the malfunction of sensor, e.g., low battery and calibration.

The authors in [13] have presented another three perspectives for classification:

1. Fault-tolerant distributed system viewpoint, that is based on the behaviour of the failed sensor, e.g., crash and omission.
2. Duration viewpoint that classifies faults based on their duration e.g., permanent and intermittent.
3. Components viewpoint, e.g., functional and informational faults.

Several fault detection techniques have been developed for sensor networks. However, the techniques were mainly designed for *time-driven*, *continuous-valued* and *homogeneous* sensors, e.g., temperature sensors. Thus, those techniques are not suitable for the *event-driven*, *binary* and *heterogeneous* nature of sensors that are needed for the ambient assisted living, e.g., motion detectors, contact sensors, etc. [14].

In an AAL system, a sensor failure is considered to be a fault from the perspective of the whole AAL system. There are two main categories of sensor failures in the AAL terminology:

- A *fail-stop failure* means that the sensor has stopped responding.
- A *non-fail-stop failure* indicates that the sensor is still responding, however, the reported values are no longer representative of the measured variable, nor the occurring events in the surrounding environment that are intended to be detected.

Sensor failures can also be classified as single-sensor failures and mutliple-sensor failures. In research works considering single-sensor failures, it is assumed that only one sensor can fail at a time [14].

In the field of AAL, Flöck has presented an overview of the binary sensors malfunctions that were observed during practical AAL implementation, e.g., faulty activation of motion detectors by sunlight, bouncing of contact sensors, and switch-off delays of motion sensors [15]. Also, Rahal et al. have reasoned the false information sent by binary sensors to be either due to an intrinsic error, e.g., the sensor’s error rate, or due to an external error, e.g., an air draft or a pet may close the door triggering false events [16]. Different types of non-fail-stop failures have been stated in the research papers. Examples of the non-fail-stop failures are:

- *Moved-location failure*, which occurs due to moving furniture that have sensors installed on it to a different area or re-mounting in the wrong location.
- *Obstructed-view failure* that occurs due to covering the sensors or its dislodgement that may result from regular use, cleaning, other non-residents, etc. [17,18].

A set of guidelines and principles for the deployment of large-scale residential sensing systems was proposed in [19], summarizing the experience gained from installing over 1200 sensors in over 20 homes to monitor human activity. The main failure modes were examined to identify the longest acceptable time interval of inactivity for each sensor. For each periodic sensor, the interval is set to 5 times the sampling period, while for event-driven sensors, it is set to 36 h. The root cause of failure is identified based on the set of simultaneous sensor failures, where the considered causes of failure are wireless link loss, dead battery, disconnected plug, sensor sub-system down, internet-down, power outage, and gateway down. The described failure detection and classification approach was applied on four deployments for seven months. The analysis of the results showed that sensors are 2.3 times more likely to fail due to being unplugged than to dead battery and that wireless link loss is a less cause of failure than the other sources of sensor down time. Failure of an entire sensor sub-system appeared to be the most common cause of failure. This performed failure analysis enabled the authors to present guidelines that could avoid some of the pitfalls and failures observed in the deployments. However, a fault detection and diagnosis system still needs to be implemented to deal efficiently with sensor failures.

The following is the most common terminology found in the surveyed literature for the evaluation of various systems;

- *true positives* (*TP*) are the data points reported as positive when they actually are positive
- *false positives* (*FP*) are the data points reported as positive while they are actually negative
- *false negatives* (*FN*) are the data points reported as negative while they are actually positive
- *true negatives* (*TN*) are the data points are reported as negative while they are negative
- *precision* measures the percentage of true positives from the total points reported as positive (TP/(TP+FP))
- *recall* measures the percentage of true positives from the actual positive points (TP/(TP+FN))
- *accuracy* measures the percentage of true positives and negatives from the data
  ((TP+TN)/(TP+TN+FP+FN))
- *failure detection latency* is the amount of time taken to detect a sensor failure after its occurrence.

**Figure 1:**
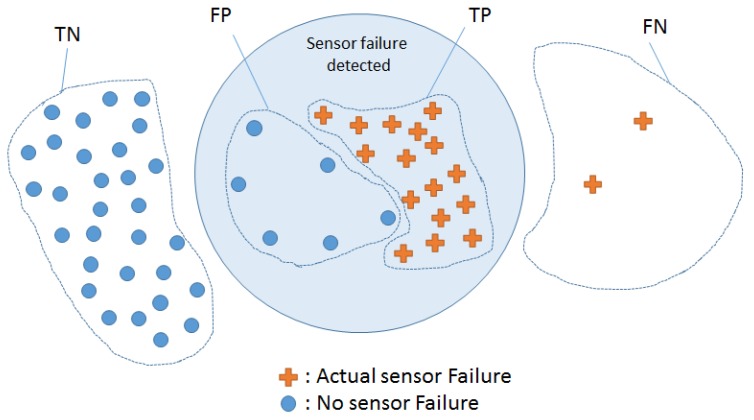
Evaluation metrics terminology for sensor failure detection system.

Figure 1 elaborates the terminology with respect to sensor failure detection systems, where the *accuracy*, *precision* and *recall* values are 85%, 72% and 88%, respectively. The *accuracy* would still be relatively high if the system does not report as many sensor failures as before (lower *TP* and higher *FN*), however, the *precision* and *recall* would significantly drop. Thus, only using the *accuracy* for evaluating the system performance is insufficient. The *precision* indicates the ratio of the correctly reported sensor failures to all the positively reported sensor failures, while the recall indicates the ratio of correctly reported sensor failures to the positive sensor failures ground truth.

## 3. Datasets

This section presents an overview of the publicly available datasets that were used in a number of the reviewed research works. Other publicly available datasets exist for ambient assisted living, but they have not been used in research papers that focus on fault detection nor fault tolerance. It is worth noting that to the best of our knowledge, all the public datasets do not include any labels of the faulty sensors data.

### 3.1. Kasteren Datasets

Tim van Kasteren has collected benchmark datasets (called house A, B and C) [20] from three single-resident apartments which were collected over 14, 23 and 19 days, respectively. Wireless sensors that gives binary output were installed; reed switches for the doors and cupboards, pressure mats for couches and beds, mercury contacts for drawers, passive infrared (PIR) sensors to detect motion of resident in different areas of the apartments and float sensors for toilet flushing detection. The number of sensors installed in the three apartments (A, B and C) are 14, 23 and 21, respectively. During the collection of data, the resident performed his daily routine freely in an unscripted manner (i.e., the resident was not told what to do or which activity to perform). Annotation of the start and end of activities was performed by the resident using handwritten activity diary or a bluetooth headset [21]. The following data is recorded in the dataset files; start and end date/time of sensor activation, sensor ID, start and end date/time of activity and activity label.

### 3.2. CASAS Datasets

The CASAS research group in Washington State University (WSU) has made 64 datasets publicly available [22]. The recorded datasets were either collected from the WSU smart apartment equipped with around 90 sensors, residential apartments that has a number of sensors that ranges between 30 to 50 sensors or SHib partner lab equipped with 25 sensors, for a duration ranging from hours to years, for single- or two-resident apartments. Some of the experiments were scripted, e.g., adlnormal data and adlinterweave data, and others were unscripted, e.g., aruba data and kyoto data. Examples of sensors installed in the apartments are motion sensors, magnetic sensors, water flow sensors, item presence sensor, stove burner sensor and temperature sensors. The following data is recorded in the datasets files; data/time, sensor ID, sensor value/status. Some of the datasets have labels for the start and end of the performed activities.

### 3.3. Placelab Datasets

Three datasets (PLIA1, PLIA2 and PLCouple1) were collected from Placelab living lab [23] (note that the Placelab dataset website has been down for months). The living lab is an apartment where volunteers live during the data collection process. Two datasets were collected from single residents for 4 h who were asked to perform a set of activities, and the third one was collected from a couple who lived freely there performing their own daily routines for 10 weeks. The datasets were annotated with the performed activities using video recordings. The apartment is equipped with around 400 sensors that range between reed switches, light sensors, motion detection sensors, water flow sensors, temperature sensors, humidity sensors, electrical current flow sensors, gas sensors, etc. [24].

### 3.4. Tapia Datasets

Emmanuel Munguia Tapia has conducted experiments for two weeks in two single-resident apartments (subject 1 and subject 2) equipped with 77 and 84 sensors, respectively. The sensors are reed switches attached to the everyday objects, e.g., drawers, doors, containers, refrigerator, etc. The residents carried out their daily activities without any scripts [25]. The following data is recorded in the datasets; activity label, start and end date/time of activity, sensor ID, start and end date/time of sensor activation.

## 4. Literature Survey Methodology

In order to conduct the literature survey, the title, abstract and keywords fields were searched in Scopus, IEEExplore, Web of knowledge and ACM databases for the following combination of terms; ("fault detection" OR "sensor failure") AND ("smart home" OR "ambient assisted living"). Scopus and Web of knowledge databases produced the largest number of relevant articles. The search was then extended on Scopus and Web of knowledge to include more combinations of the keywords shown in Table 1, so that the combination is as follows; ((Group A AND Group C) OR Group D) AND Group B. Only the papers concerned with non-intrusive ambient binary sensors were included in the survey. The obtained articles were cross-referenced, and a total of 30 papers were selected for the review. It was observed that these 30 papers were all published between 2008 and 2017.

The main focus of the research works can be mainly categorized as works concerned with:

- sensor failure detection in AAL
- fault-tolerant ADL recognition
- fault-tolerant abnormal behavior detection
- fault-tolerant indoor localization system/location tracking
- maintenance scheduling/management
- fault detection and diagnosis framework for AAL

The reviewed papers classification is shown in Table 2. These papers are presented and analyzed in detail in the next section.

## 5. Literature Survey Results

This section provides a state-of-the-art review for the sensor failure detection systems and fault tolerance methods in the presence of sensor failures in AAL systems equipped with *non-intrusive*, *binary*, *event-driven* sensors. The research works are categorized according to the function of the proposed systems as well as the approach that their methods are based on: correlation-based fault detection, model-based fault detection, fault-tolerant location tracking, fault-tolerant activity recognition or fault detection and diagnosis framework for AAL, respectively. A glossary of the technical terms can be found at the end of this paper.

### 5.1. Correlation-Based Fault Detection

The following research papers proposed sensor failure detection systems based on either sensor-appliance, sensor-sensor or sensor-activity correlations.

FailureSense [17] was presented by Munir and Stankovic to detect fail-stop and non-fail stop mutliple-sensor failures. It is based on exploiting the correlation between the trigger of motion sensors and the activation/deactivation of electrical appliances. The correlation is represented by the smallest interval of sensor firing after and before a turn on/off event within 5 min, denoted by $I_{A}$ and $I_{B}$, respectively. The distribution of $I_{A}$ and $I_{B}$ is modelled by Gaussian mixture model (GMM), whose parameters are estimated from the training data using the expectation maximization (EM) algorithm. Online failure detection takes place by monitoring the sensor appliance behaviour represented by $I_{A}$ and $I_{B}$. A failure is reported when a deviation occurs in the distribution beyond predefined thresholds for each sensor-appliance pair. The thresholds are computed using the training dataset. Evaluation was performed on three real-home datasets with around two thirds of the dataset used for training and one third for testing. Fail-stop failure was simulated by removing all the readings of a sensor after its randomly assumed day of failure. For the obstructed-view failure, simulation took place for two of the homes by randomly removing a 10-day period during which sensor view is considered to be obstructed, and for the third home, physical obstruction of the view of 5 motion sensors was done during the data collection phase. Simulation of the moved-location failure was done by replacing the readings of failed sensor with the readings recorded by the sensor at the newly moved location. The evaluation metrics used are the precision and recall of failure detection, where they represent the percentage of the true failure alerts from the total observed failure alerts, and the percentage of the true failure alerts from the sensor failures, respectively. Experiments of the fail-stop, obstructed-view and moved-location failures produced approximately 82.8%, 90.5% and 86.8% average precision, with an average recall of 92.86%, 84.4% and 89%, respectively. The effect of increasing the number of sensors that experience fail-stop failures on the percentage of failure detection has been also examined, showing an average of 86.6% sensor failure detection. On the other hand, a limitation of the proposed approach is that the average median failure detection latency is 22.08 h.

Ye, Stevenson and Dobson presented a technique to detect missing data in event-driven sensors based on temporal correlation and time-series analysis [26]. Temporal correlation relationship is defined to indicate if two sensors fire within a preset time interval. A missing data is reported when one of two highly correlated sensors fires without the other. For each sensor, the next firing time is predicted using non-linear time analysis technique, and if it does not fire at the predicted time, then it is considered as missing data. Evaluation is carried on Kasteren dataset [20] (house A), in which randomly chosen sensors events were removed from the testing data, using precision and recall metrics for each of the temporal correlation and time series approaches independently, then combined. The effect of changing predefined parameters of the algorithm on the performance was also examined. Moreover, the relation of increasing the error rate percentage (percentage of data removed) in the testing set on precision and recall was plotted along with increasing the percentage of training set. The results on the examined dataset have shown that the performance of using the temporal correlations for detecting missing events is better than using the time-series analysis. Also, it was observed from the results that using both temporal correlation and time-series analysis simultaneously for failure detection had a very low impact on the performance improvement. Using temporal correlation with data split by half for training and testing sets, the precision was nearly 70% and the recall decreased from around 80% to 40% with increasing the error rate from 10% to 90%. Increasing the training data to 90%, has made the precision to be around 78% and the recall to decrease from 85% to 75%. The authors stated that the proposed approaches could not be sufficiently evaluated on the chosen dataset, as it has few sensors and is collected over a short duration.

Kodeswaran et al. aimed to propose a system called Idea, for monitoring the activities of daily living while preserving a reduced maintenance overhead [27]. It is based on the assumption that there are redundant heterogeneous sensors installed for detecting each activity. Maintenance is scheduled according to the impact of a sensor failure on the performance of the system to detect ADL. The main components of Idea are; ADL signature Extraction, ADL detection, Impact estimation, Sensor Failure detection and Maintenance scheduling. Frequent itemset mining algorithm is used to form a rule-base containing the frequently occurring subsets of sensors for each ADL, and then the most probable time of day of occurrence and duration of activity are calculated from the training dataset. The critical sensors are identified based on their impact on detecting the ADL, which depends on the redundancy level per ADL using the training dataset. For critical event-driven sensors, a failure alert is flagged if the time elapsed since the last detection of ADL exceeded a threshold. For non-critical sensors, a rarity score is computed as the probability that a sensor has not been triggered while certain ADL, that should involve this sensor, has occurred. Experiments were conducted on Kasteren [20] (house A, B and C) and CASAS [22] datasets (aruba, twor9-10, twor2009, tworsmr and adlnormal) using 80% of the dataset for training. The accuracy of ADL detection was investigated in the presence of fail-stop sensor failures, emulated by discarding all the events of the failed sensor, and compared to Naive Bayes classifier (NB) and Hidden Markov model (HMM) algorithms. The maintenance efficiency was also evaluated in terms of the number of maintenance visits and per-home maintenance inter-arrival times. Across all the datasets, the ADL detection accuracy is reduced in average by approximately 0.5%, 1% and 3% in the presence of 1, 3 and 7 failed sensors.

Dealing with sensor faults in smart homes using data-driven approach was proposed by Monekosso and Remagnnino [28]. The proposed method aimed to detect sensor faults, mask it, and differentiate between anomalous activities and sensor deviation by combining reconciliation with failure detection techniques. The approach has two components; one component deals with random measurement fluctuations using data reconciliation, while the other component deals with systematic deviations due to sensor failures or anomalous activities. Models of sensors correlations are built using historical data via principle component analysis (PCA) and canonical correlation analysis (CCA). The models are refined continuously and can deal with heterogeneous sensors types to be used for detecting sensor faults. Experiments were carried out using Kasteren dataset [20] (house A). Two case studies were implemented by injecting intermittent and permanent faults into the dataset. A permanent fault was simulated on a sensor by removing its readings from the testing dataset after the assumed failure point of time. A transient sensor fault was injected by corrupting random instances of sensor readings with wrong values.

An approach for data-driven failure detection based on clustering was proposed by Ye, Stevenson and Dobson. They address non-fail-stop sensor failures as a clustering-based outlier detection problem [18,29]. DBSCAN clustering based outlier detection algorithm is used. The similarity between binary sensor events is calculated using least common subsumer (LCS) based on their semantic features; time stamp, the object to which a sensor is attached, location and user. Data points are clustered into groups and then the groups are sorted by their size in descending order. Shoulder-location method is used to select the threshold below which a cluster is considered small. To each data point, a cluster-based local outlier factor (CBLOF) is assigned which is a function in the size of the cluster to which this point belongs, the similarity between the point and the closest large cluster, and the historic faulty sensor behaviour. A point is considered as an outlier if its CBLOF is below a threshold defined by the shoulder location method. The technique was evaluated on Placelab [23] (PLCouple1), Kasteren [20] (house A and B) and CASAS [22] (adlinterweave) datasets with injecting random and systematic anomalies. Random abnormal events were injected into the datasets by randomly creating new sensors events within randomly selected time slots. While systematic abnormal events are injected by selecting random sensors and creating an event for each of the selected sensors within each time slot of the testing data. Plots of the precision and recall against the injection rate of abnormal events were presented.

In another attempt, detection of sensor failures was tackled using classification. Kapitanova et al. proposed simultaneous multi-classifier activity recognition technique (SMART) [14,30], which uses top-down application level semantics to detect non-fail-stop single-sensor failures. Furthermore, the research work addresses schedule maintenance according to failure severity and improvement of activity recognition accuracy in the presence of failures. Multiple classifier instances are trained offline by excluding each time a sensor out of the training set resembling a sensor failure, and one time with all sensors present in the set. Online detection of a fault is achieved by assessing the relative performance of the classifiers that has a missing sensor versus the one trained with all sensors, thus a fault is detected and identified. Severity analysis is performed to evaluate the impact of sensor failure on the accuracy of activity detection. As the level of sensor redundancy increases per activity, the urgency of repairing a faulty sensor decreases. Fault-tolerance of the activity recognition is achieved by updating the classifier ensemble with the classifiers that were previously trained to deal with a particular sensor failure. The system was evaluated using CASAS [22] and Kasteren [20] (house A and B) datasets considering only prepare breakfast, lunch and dinner activities. NB and HMM classifiers were used. Stuck-at failures and misplacement failures were introduced manually to the datasets. To simulate stuck-at failure, the value of the failed sensor is set to 1. For simulating misplacement failure, the data of failed sensor is replaced with the sensor in its new location. The results showed that this approach could decrease the number of maintenance dispatches by 55%, identifies non-fail stop failures by 85% accuracy, and improve activity recognition accuracy in presence of sensor failures by 15%.

### 5.2. Model-Based Fault Detection

The following researchers have used model-based fault detection based on localization systems. An indoor human localization (IHL) system with fault detection focusing on hardware as well as human-made single faults was presented by Veronese et al. [31]. The IHL system consists of three main components; an RF-based localization subsystem, an off-the-shelf modular wireless home automation subsystem and a fault detection subsystem. The types of sensors chosen for home automation were contact sensors and passive infrared (PIR) sensors. A model-based fault detection approach was applied based on the concept proposed by Isermann [48] which states that a fault can be detected using the dependencies between different measured signals. The activation of the home automation sensors and its features were used to estimate the resident’s location. Also, the position of the resident is estimated independently with the localization subsystem. The fault detection subsystem compares the two estimated location areas, and flag a fault whenever there is no intersection between the two areas. Experimental work was done, where 19 fixed LAURA anchors and 7 Z-wave devices were fixed across the rooms of the university building. Two fault scenarios were considered; forgotten worn device and blinded PIR motion detector. The results showed that the faults could be detected using the proposed approach. As a continuation of the work, multi-user simulation was conducted using three virtual users trajectories, the faults could be detected in the presence of multiple users with specificity and sensitivity above 90% [32].

Danancher proposed model-based location tracking of single as well as multiple inhabitants in smart homes [10]. He treated the location tracking of inhabitants as a problem of discrete event system modeling. Finite automata was used to model the observable motion of inhabitant, where each state represents a zone in the apartment, each event represents the rising or falling edge of binary sensor, and each transition is the observable location change. A case study was presented for an apartment equipped with motion detectors and door barrier sensors. The impact of sensor faults on the performance of location tracking was discussed. The applicability of three model-based fault detection and isolation (FDI) approaches; diagnoser, template and residual approaches, were investigated for fault-tolerant location tracking. An adaptation to the residual-based approach was applied to a case study of tracking a single inhabitant. Three fault scenarios were considered; spurious activation of a motion sensor, failure of power supply of door barrier sensor and a failure of motion detector sensor. The approach could not detect nor isolate faulty sensors in the proposed faulty scenarios. The author concluded that the industrial FDI approaches are not suitable for sensor faults in smart home and that a new FDI approach designed specifically for smart homes should be developed.

Another discrete event system approach for location tracking was proposed by Wu et al. [49]. The motion of the resident is modeled using an automaton model and the observations of motion events from sensor signals are described using the state tree of Graph theory. An Observer is then used to estimate the location of the inhabitant. Dealing with transient sensor faults is performed by adding a reset procedure to the state tree and the observer so that they return to the initial state whenever blocking occurs due to missing or disordering of a sensor event. This ensures that the location tracking returns to output correct estimation results after deviating due to the transient sensor fault. However, false location estimation still occurs. A scenario of the motion of inhabitant in the presence of a missing sensor event was presented.

Amri et al. have proposed fault detection approach for indoor localization based on set-membership fault detection using the q-relaxed intersection method [36]. The random walk model is used as the mobility model of the resident. The PIR sensor activation leads to the activation of a box representing its coverage area. At one second time step, the measurement boxes are observed and the predicted boxes are deduced using the mobility model. The q-intersection method deduces the location zone of the resident using these boxes. Outlier detection takes place by comparing the solution set obtained and the measurements. Experiments were conducted in a living lab equipped with PIR sensors.

### 5.3. Fault-Tolerant Location Tracking

A fault-tolerant location tracking system was presented by Rahal, Pigot and Mabilleau, which aims to localize single inhabitant using the already installed sensors in smart home [16]. The authors aimed to provide a reliable location tracking system that can estimate the location of inhabitant accurately despite the false trigger of sensors that may occur due to various factors. The adopted approach is based on sensor fusion, in which particle filters approach is used to estimate the new inhabitant’s location using the last known position and the last sensor event. To evaluate the system, experiments were conducted in the DOMUS apartment, where non-intrusive unobtrusive sensors (infrared (IR) presence sensors, tactile carpets, smart light switches, contact sensors and pressure detectors) are installed. A daily routine scenario was performed by 14 subjects, one subject each time, and the results showed an accuracy in location tracking above 85%. The system performance was also investigated with respect to the inhabitant’s profile, sensor configuration, inhabitant’s dynamics and in the presence of noise. The results showed that the accuracy of the system is profile-independent. The accuracy of localization when using only infrared sensors is similar to using all the sensors. However, the IR sensors are more prone to false triggers, thus, the authors recommended the usage of at least one other type with IR sensors. The system accuracy remained at 84% when 2.5% and 5% noise were applied to the collected data.

A similar system was proposed by Ballardini et al. that is based on estimating the resident location in the presence of false positive or false negative sensor readings via Bayes filtering [46]. The system uses a probabilistic model of the sensors and a motion model of the inhabitant. The proposed approach was tested on two noisy datasets that use PIR sensors (observed frequent false triggering of a motion sensor when no person is moving, and trigger of atrium’s motion sensor when motion occurs in the dining room), producing 5% and 9% error rates in localization.

A fuzzy set-based approach for localization tolerating sensor failures was proposed by Ahvar et al. [47]. The approach relies on using several functionally redundant sensors at specific nodes. The system is composed of sensor nodes and context broker based on the fuzzy set theory. The apartment is divided into zones and equipped with various types of ambient sensors. The sensors send context information, then the membership values for each zone is computed. The highest value indicates the user location. A case study was presented and simulated using the DPWsim simulator with different sensor error rates. However, the system was not verified using a real dataset.

### 5.4. Fault-Tolerant Activity Recognition

In addition to the fault-tolerant activity recognition implemented by SMART system [14,30] and Idea system [27], a framework of fault-tolerant activity recognition was addressed by Hong et al. [38,39,40]. First, the effect of sensor failures on the accuracy of activity recognition was investigated. Only binary sensors were considered for monitoring the ADL in smart homes. Sensor evidence reasoning network was designed based on activity hierarchy of ontology for activity recognition while tolerating uncertainty in the sensors’ measurements. The discounting values depend on the manufacturer statics on the sensors. To validate the proposed approach, a case scenario was presented. In addition, sensors data recordings were collected from smart laboratory environment of a kitchen area for four weeks, and then, offline analysis was performed to verify the sensor data with video recordings. The sensor data was fed to the evidential reasoning network that is based on the Dempster-Shafer theory. The performance of activity recognition was assessed with respect to the number and combinations of sensor failures. Mckeever et al. [41] have extended the evidence of theory to incorporate temporal features and evaluated their proposed framework on Kasteren dataset [20] (house A). A limitation of the approach is that expert knowledge is needed for the sensor mass functions and sensor quality. Also, knowledge from users is used to get information about the temporal features of activities.

Liao et al. [42,43,44] have proposed an activity recognition framework that deals with uncertainty in sensor measurements based on Dempster-Shafer theory of evidence while considering the effect of historical information and activity patterns. This is implemented through a framework with a lattice structure, which has a context layer that includes combinations of sensors derived from the historical data of inhabitant. Two types of uncertainty sources were considered; sensor hardware and context uncertainty due to the variability in human activities. A case study was presented in addition to applying the proposed approach to a publicly available dataset (Tapia dataset, subject 1) [25] collected from an apartment equipped with binary sensors. The performance was evaluated using precision, recall and F-measure metrics for activity recognition.

A Weighted Dempster-Shafer theory was presented by Javadi, Moshiri and Yazdi [45], where a weight for each sensor is assigned based on the historical data and activity patterns of the resident. In the training phase, 10 models are built for each sensor, and then in the testing phase, a weight for each sensor is calculated based on the membership degree of each sensor signal to the sensor’s models. The proposed method is applied to a dataset (Tapia dataset, subject 1) [25] and evaluated through the accuracy detection rate of toileting activity. A drawback in the experiments is that, sensor faults were not injected to the dataset.

Abnormal behaviour recognition in the presence of sensor failures/uncertainties was addressed by Marhic et al., it is based on the evidential approach using transferable belief model (TBM) [37]. It is assumed that there are three or more heterogeneous redundant sensors per each monitored activity. The system consists of Sensor FDI and the Abnormal behaviour detection modules. The Sensor FDI analyses the conflict between the heterogeneous redundant sensors using sensor fusion calculated by the Smet’s operator and two experts. Abnormal behaviour is then detected by comparing the normal behaviour of inhabitant represented by the Markov chain model (MCM) and the detected/predicted behaviour within the TBM framework. Experiments were conducted on datasets collected from performing sitting, lying and standing activities with various single sensor failures, during which pressure sensor, omni-directional vision sensor and an accelerometer were used. The authors showed the ability of the system to detect abnormal behaviour in presence of sensor failures (unplugging sensor for a period of time) and highlighted some limitations that could be addressed in the future.

Methods for fault tolerance in Ambient Assisted Living were suggested by Ahvar et al. [50]. Data from binary sensors, e.g., movement sensors, may be corrected using a model of the inhabitant behaviour. While fault tolerance for analog data from sensors, e.g., temperature sensors, may be implemented using sensor fusion. However, the system was not verified against faults in a case study.

### 5.5. Fault Detection and Diagnosis Framework for AAL

A fault detection and diagnosis framework for Ambient Intelligent systems was presented by Mohamed, Jacquet and Bellik [33,34], however, it is concerned only with the tasks performed by the systems through the actuators. The approach is based on modeling the physical phenomena that are supposed to occur in the environment due to the activation of a particular actuator. The system then automatically creates links between actuators and sensors at run-time using the models. It predicts the expected sensor reading due to the activation of an actuator and compares it with the actual sensor reading to detect if a fault has occurred. Simulations were performed to illustrate the operation of the system and show the ability of the system to discover new components at run-time. The basic idea of the diagnoser model was presented without details.

A self-diagnosis framework was proposed by Oliveira et al. [35], where a Bayesian network construction algorithm is used to create a Bayesian network for each scenario that is supposed to be fulfilled by the AAL system to assist the user. The algorithm takes as inputs the rules file that specifies the causal relations between variables, and the scenario description file that specifies the required assistance and the home description. Conditional probability distribution is calculated for each child node. The real values are then compared with the predicted ones and a fault is flagged if the readings are different. Using the causality relations and conditional rules, a diagnostic is reached. A case study was investigated to show the ability of the proposed framework to detect and diagnose faults. However, like the previous system [34], the framework would only work fine for the tasks that involve a sensor-actuator feedback.

## 6. Discussion

### 6.1. Correlation-Based Fault Detection Systems

Next, we discuss the pros and cons of the correlation-based fault detection approaches.

FailureSense [17] has good average precision and recall for the examined fail-stop, obstructed-view and moved-location failures. Also, the experiments show consistent performance for failure detection with increasing the number of sensors that had fail-stop failures. However, the method does not work well if the failed sensor is not associated with any electrical appliance. In addition, its average failure detection latency is not suitable for emergency situations. Another limitation of the system is that, it is based on the assumption that the resident has to be physically beside the appliance to turn it on. In addition, the system performance depends on the behaviour of residents (i.e., the residents turn on/off electrical appliances remotely or their behaviour pattern in using electrical appliances).

Using temporal correlations and/or time-series analysis in [26] only relies on sensors firing to detect missing sensor events. The temporal correlation method achieves better results than using time-series analysis. However, the average precision and recall on the examined dataset with random non-fail-stop failures are not as good when increasing the error rate percentage, except when the training data percentage was increased to 90% . This makes the performance of the proposed method still questionable and needs to be evaluated on other larger datasets.

The approach of the Idea system [27] is designed to suit homes equipped with functionally redundant sensors per activity of daily living. Otherwise, it will not work as expected. In this work, only fail-stop failures were considered. The reduction in the ADL detection accuracy in the presence of sensor failures is relatively low. Thus, an efficient fault tolerant activity recognition seems to be promising using the proposed approach. However, the effect of monitoring multiple ADLs to detect sensor failures on the failure latency detection, and the effect of rarity threshold on the false positive alerts were the only assessments used for the sensor failure detection subsystem. Those assessments are not enough to be able to see the efficiency of the sensor failure detection. Also, non-fail-stop failures need to be considered in the experiments. In our opinion, detecting failures using time elapsed is not an efficient solution and using the rarity score assumes that the system has not misclassified the activity. Similarly, the detection of sensor failures using the proposed approach in [28] was not thoroughly evaluated. The experiments were only concerned with the ability of the system to detect and isolate a faulty sensor, without any quantitative evaluation of the performance. Another drawback is that the injected faults in the experiments were applied on only a single sensor.

The advantage of using clustering approach as in [18,29], is that no training phase is required. However, the proposed method aims to detect false sensor triggers, but it can not detect missing sensor data. Another limitation is that the failure detection takes place in a post-processing step on the collected data. Also, the false positive trigger is less likely to be detected if it is associated with a sensor that has similar features to other correctly working sensors. Using multiple classifier instances [14,30] produced promising results for sensor failure detection and fault-tolerant activity recognition. However, the disadvantage of this approach is that the training effort is large and it increases proportionally with the number of installed sensors, thus the system is non-scalable.

### 6.2. Model-Based Fault Detection Systems

The reviewed model-based fault detection systems do not seem to provide better results than the correlation-based fault detection systems. The approaches mainly rely on checking if the sensor trigger is consistent with the predicted location of the resident. The method proposed in [31] that uses RF-based localization system in addition to the environmental binary sensors installed at home, can not identify if the fault source is the localization system or the installed sensors. In another research work [10], applying residual-based fault detection to the location tracking finite automata model of an inhabitant could not detect nor isolate the faulty sensors. Only preventing the transient sensor faults from blocking the discrete event location tracking model was proposed in [49]; however, it was not even capable of tolerating those faults. In [36], comparing the motion sensors triggers with the random walk mobility model is not reliable, since this mobility model can produce unrealistic patterns as it does not keep track of the past locations and speed.

### 6.3. Fault-Tolerant Location Tracking Systems

The fault-tolerant location tracking systems reviewed are based on attempting to estimate the location of the resident under uncertainty of sensors whether through sensor fusion [16,46] or fuzzy theory [47]. The results seem promising, however, the systems need to be investigated more thoroughly in real-time experiments.

### 6.4. Fault-Tolerant Activity Recognition Systems

In addition to the SMART [14,30] and Idea [27] systems discussed before for proposing sensor failure detection and fault-tolerant activity recognition, fault-tolerant activity recognition based on recognizing activities under sensors uncertainty were reviewed. The works that used the evidence theory [37,38,39,40,41,42,43,44,45] have the disadvantage of requiring lots of expert knowledge.

### 6.5. Fault Detection and Diagnosis Framework

The reviewed fault detection and diagnosis frameworks [33,34,35] were designed to only suit AAL systems involved with sensor-actuator feedback.

## 7. Conclusions

In the last 10 years, an increasing interest in tackling sensor failures/faults in AAL has been observed. However, there is still much to be done in this area to offer a dependable system for the users.

Table 3 and Table 4 summarize the work reviewed in Section 5. For each research work; the contributed system, its method, algorithm(s), experiments conducted and performance metrics used are listed in this table.

The overall general limitations of the existing works can be categorized as follows:

Limitations regarding the approaches:

- Most of the existing works have developed their approaches considering only single failures. However, it may happen that more than one sensor fail simultaneously.
- The majority of the developed algorithms use parameters or thresholds that need to be chosen by an expert rather than being deduced automatically.
- Differentiating between failed sensors and anomalies in human behaviour is still a challenge that needs to be addressed.

Limitations regarding the datasets:

- The public datasets used for the training and testing phases are limited to short duration, low sensor node redundancy and single resident apartments.
- Also, the data in the publicly available datasets was originally collected for activity detection with labelled activities, thus, failures or anomalies were not labelled. Instead, sensor failures were manually injected and simulated by the researchers, which may not be representative of real-home sensor failures rate and percentage.

Limitations regarding the experimental methodology:

- It is difficult to compare between the efficiency of the presented approaches because not all the authors use the same evaluation criteria and same testing data. Thus, there is a need for standardized evaluation criteria.
- Beside the accuracy, precision and recall, the sensor failure detection latency is an important criterion to be considered.
- Real-time online evaluation of the algorithms was not carried out, instead the data collected from previous experiments or datasets were fed to the algorithms.
- The proposed approaches should additionally be evaluated on data collected from elderlies with physical and/or cognitive deficiencies.

As illustrated by the number and importance of the limitations of the existing works, fault-tolerance in AAL is still in its early phase. Thus, intensive research work is still needed to tackle them. The research topics to be addressed can be grouped in the three following research questions:

- Can novel machine learning techniques tackle the problem of sensor failure detection in AAL without the need for expert knowledge?
- Should the research priority be directed towards enhancing the accuracy of binary sensors or instead towards dealing with the faulty sensors data through fault-tolerant systems?
- Would differentiating between behaviour anomalies of residents and sensor anomalies be possible?

As a conclusion, as highlighted by this systematic literature review, methods for fault-tolerant Ambient Assisted Living are still in their infancy stage. Also, intensive research works would be needed to ensure the development and implementation of a robust sensor fault detection and diagnosis system for Ambient Assisted Living in a near future.

## Figures and Tables

**Table 1:** Search keywords.

Group A ^1^	Group B	Group C	Group D
"sensor*"	"smart home"	"fault detection"	"sensor* error"
	"ambient assisted living"	"failure detection"	"sensor* failure*"
	"AAL"	"fault toleran*"	"sensor* fault*"
	"location tracking"	"fault identification"	"sensor reliab*"
	"actvity recognition"	"failure identification"	"faulty sensor*"
	"activity monitoring"	"fault diagnosis"	"*reliable sensor"
	"activity detection"	"FDI"	"uncertain sensor"
	"home* based care"	"fault isolation"	"sensor diagnos*"
	"indoor localization"	"fault prevention"	"sensor node fail*"
		"fault prediction"	"fail* sensor*"
		"fault recover*"	"anomal*" AND "binary sensor*"
		"self-check*"	
		"self-heal*"	
		"dependable"	
		"failure management"	

^1^ * replaces any number of characters, i.e., sensor* will search for sensor, sensors, sensory, etc.

**Table 2:** Main focus of the research works.

Focus	Research Work
Sensor failure detection	[10,14,17,18,26,27,28,29,30,31,32,33,34,35,36]
Maintenance scheduling/management	[14,27,30]
Fault-tolerant ADL recognition	[14,27,30,37,38,39,40,41,42,43,44,45]
Fault-tolerant abnormal behavior detection	[37]
Fault-tolerant indoor localization system/location tracking	[16,46,47]

**Table 3:** Summary of the reviewed work in Section 5.1 and Section 5.2.

Source	Contribution	Method	Algorithm	Experiments		Performance Metrics
				Data	Failure Type	
[17]	sensor fault detection	sensor-appliance correlations	GMM & EM	custom datasets	injecting fail-stop and non-fail-stop (obstructed-view and moved-location) failures	precision, recall & failure detection latency
[26]	sensor fault detection	sensors correlations	mutual information and non-linear time series analysis techniques	publicly available dataset (Kasteren, house A)	injecting non-fail-stop failures (removing random sensors events)	precision & recall
[27]	sensor fault detection, fault-tolerant activity recognition & maintenance scheduling	sensor-activity correlations	frequent itemset mining algorithm & rarity score calculation	publicly available datasets (Kasteren; house A, B & C, and CASAS; aruba, twor9-10, twor2009, tworsmr & adlnormal)	injecting fail-stop failures	sensor failure false alert rate, failure latency detection & reduction in ADL detection accuracy in presence of failures
[28]	sensor fault detection and masking	sensors correlations	PCA & CCA	publicly available dataset (Kasteren, house A)	injecting permanent and intermittent faults (i.e., fail-stop and non-fail-stop)	ability to detect faults
[18,29]	sensor fault detection	clustering-based outlier detection	DBSCAN clustering algorithm	publicly available datasets (Placelab, PLCouple1, and Kasteren; house A and B, and CASAS, adlinterweave)	injecting random and systematic false positive sensor triggers (non-fail-stop)	precision & recall
[14,30]	sensor fault detection, fault-tolerant activity recognition & maintenance scheduling	simultaneous use of multiple classifiers	NB, HMM, hidden semi-Markov model (HSMM) & decision trees	publicly available datasets (Kasteren, house A and B, and CASAS (not specified))	injecting non-fail-stop failures (stuck-at and moved-location)	failure detection accuracy & failure latency detection
[31,32]	indoor localization system with fault detection	model-based fault detection using RF-based localization & home automation subsystems	estimating the location using the activation of home automation sensors and the RF-based localization subsystem	custom dataset	collected with blinded PIR sensor and forgotten worn device	sensitivity & specificity
[10]	location tracking with sensor fault detection	model-based fault detection using a model of the observed motion of the inhabitant	finite automata & residual calculation	scenario of motion of inhabitant	in the presence of fail-stop and non-fail-stop failures	ability to detect faults
[49]	location tracking dealing with transient faults	state estimation with reset procedure	automaton model & state tree of graph theory	scenario of motion of inhabitant	scenario of the presence of missing sensor event (non-fail-stop)	location estimation in presence of transient sensor faults (non-fail-stop)
[36]	localization system with sensor fault detection	model-based fault detection using the random walk model of inhabitant	set-membership fault detection using the q-relaxed intersection method	custom data collected from Living lab	not specified	ability to detect faults (outliers)

**Table 4:** Summary of the reviewed work in Section 5.3, Section 5.4 and Section 5.5.

Source	Contribution	Method	Algorithm	Experiments		Performance Metrics
				Data	Failure Type	
[16]	fault-tolerant localization system	state estimation based on sensor fusion	particle filters approach	custom data collected	injecting random sensor noise (non-fail-stop)	localization accuracy & mean belief
[46]	fault-tolerant localization system	state estimation	bayes filtering	custom dataset	data collected in presence of noise	localization error rate
[47]	fault-tolerant localization system	fuzzy-based approach using various types of ambient binary sensors	fuzzy-set theory	scenario and simulation of motion of inhabitant on DPWsim simulator	in the presence of sensor node failure fail-stop and non-fail-stop	localization accuracy
[38,39,40]	fault-tolerant activity recognition framework	evidential approach for reasoning under uncertainty	sensor evidence reasoning network & dempster-shafer theory	scenario and custom data collected	injecting different combinations of sensor failures	belief in activity inference
[41]	fault-tolerant activity recognition framework	evidential approach for reasoning under uncertainty	temporal evidence theory & dempster-shafer theory	publicly available dataset (Kasteren, house A)	no faults injected	activity recognition precision, recall & F-measure
[42,43,44]	fault-tolerant activity recognition framework	evidential approach for reasoning under uncertainty	evidential lattice structure considering historical information and activity patterns & dempster-shafer theory	scenario and publicly available dataset (Tapia, subject 1)	no faults injected	activity recognition precision, recall and F-measure of activity recognition
[45]	fault-tolerant activity recognition framework	evidential approach for reasoning under uncertainty	weighted dempster-shafer theory & fast fourier transform	publicly available dataset (Tapia, subject 1)	no faults injected	activity recognition accuracy
[37]	fault-tolerant abnormal behaviour detection	evidential approach for reasoning under uncertainty in the presence of heterogeneous redundancy per activity	sensor fusion based on Smet’s operator, experts, TBM & MCM	custom data	collected with inducing non-fail-stop sensor failure	ability to detect abnormal behaviour and/or failed sensor
[33,34]	fault detection and diagnosis framework for AAL	modeling the physical phenomena that are supposed to be detected by sensor due to the activation of an actuator	not applicable	simulating a scenario in presence of sensor failure	not specified	ability to detect system fault
[35]	self-diagnosis framework for AAL	Bayesian network for each scenario that is supposed to be fulfilled by the AAL system to assist the user	bayesian network construction algorithm	scenario of inhabitant in the presence of sensor failure	fail-stop	ability to detect system fault

## Abbreviations

The following abbreviations are used in this manuscript:

AAL	Ambient assisted living
ICT	Information and communication technologies
ADL	Activities of daily living
GMM	Gaussian mixture model
EM	Expectation maximization
NB	Naive Bayes
HMM	Hidden Markov model
PCA	Principle component analysis
CCA	Canonical correlation analysis
DBSCAN	Density-based spatial clustering of applications with noise
LCS	Least common subsumer
CBLOF	Cluster-based local outlier factor
SMART	Simultaneous multi-classifier activity recognition technique
IHL	Indoor human localization
PIR	Passive infrared
FDI	Fault detection and isolation
IR	Infrared
TBM	Transferable belief model
MCM	Markov chain model
HSMM	Hidden semi-Markov model
